# The Art of Influencing Consumer Choices: A Reflection on Recent Advances in Decision Neuroscience

**DOI:** 10.3389/fpsyg.2019.03009

**Published:** 2020-01-21

**Authors:** Nadège Bault, Elena Rusconi

**Affiliations:** ^1^School of Psychology, University of Plymouth, Plymouth, United Kingdom; ^2^Center for Mind/Brain Sciences (CIMeC), University of Trento, Trento, Italy; ^3^Department of Psychology and Cognitive Science, University of Trento, Trento, Italy

**Keywords:** value-based decisions, choice biases, marketing, regulation, decision neuroscience

## Abstract

In recent years, our knowledge concerning the neurobiology of choice has increased tremendously. Research in the field of decision-making has identified important brain mechanisms by which a representation of the subjective value of an option is built based on previous experience, retrieved and compared to that of other available options in order to make a choice. One body of research, in particular, has focused on simple value-based choices (e.g., choices between two types of fruits) to study situations very similar to our daily life decisions as consumers. The use of neuroimaging techniques has deepened and refined our knowledge of decision processes. Additionally, computational approaches have helped identifying and describing the mechanisms underlying newly found components of the decisional process. They provide mechanistic explanations for diverse biases that can drive decision makers away from their own preferences or from rational choices. It is now clear that both attentional and affective factors can exert robust effects on an individual’s decisions. Because these factors can be manipulated externally, academic research and theories are of great interest to the marketing industry. This approach is becoming increasingly effective in manipulating consumer behavior and has the potential to become even more effective in the future. Another line of research has revealed differences in the decision-making neural circuitry that underlie sub-optimal choice behavior, rendering some individuals particularly vulnerable to marketing strategies. As neuroscientists, we wonder whether relevant institutions should direct their efforts toward raising citizens’ awareness, demanding more transparency on marketing applications and regulate the most pervasive communication techniques in marketing, in view of their current use and of recent research progress.

## Attentional Biases in Consumer Choices

Tremendous progress has been realized in the last decade in our understanding of attentional effects on decision processes, through the description of their neurocomputational mechanisms. Thus, we will focus here on those mechanisms to illustrate how they can inform marketing strategies. Psychological and neural accounts of the role of memory and affective mechanisms in consumer decisions can be found in Plassmann and Karmarkar (2015).

### Cognitive and Neural Mechanisms of Simple Choice

When facing a simple decision, for instance picking a fruit to eat in a basket containing several types of fruits, our brain computes a value signal. The value represents the expected benefit of consuming the good based on previous experience. Recent cognitive models of decision-making propose that a value is assigned to all the options available, then the values are compared in order to reach a decision (Rangel and Clithero, 2014). Expected delays, potential price, or uncertainty in its obtainment of the good will all be incorporated into the value signal. How exactly the value is computed, though, is still under scrutiny. Much evidence supports the theory that values are computed through reinforcement learning. A value is updated when our experience in consuming the good does not match our expectations, a mechanism that supports adaptive behavior. This learning mechanism is implemented in the brain by dopaminergic neurons of the ventral striatum. These neurons encode a prediction error signal which serves as an update signal for the value (Schultz, 1998; Tobler et al., 2005). They project to a frontal region called the ventromedial prefrontal cortex, which is thought to store the value signal (Ruff and Fehr, 2014). However, the way we value options often depends on our internal states (e.g., how hungry we are at that particular moment) and on states of the world (we might value more a juicy fruit in the summer than in the winter). Assuming that values of goods are stored globally fails to explain why choices can vary with the decision context.

Another theory proposes that we separately evaluate all attributes of the available options and integrate them at the time of choice (Rangel and Clithero, 2014). The value of an apple is not represented as such; rather, value associated with its color, taste, smell or shape are encoded separately. Considering the attributes of a good and retrieving the values associated with those attributes requires attention.

### The Influence of Attention on Decisions

Krajbich and Rangel (2011) proposed that attention fluctuates among the different items being evaluated during a decision, and this affects the computation of their value. They applied a well-established model in perceptual decision-making (Ratcliff, 1978; Ratcliff et al., 2016) to simple value-based choices in order to characterize the link between attention – as measured by eye gaze and decision latency – to decision output through the hidden value computation process. Their attentional drift diffusion model (aDDM), applied to binary choices, states that the values of the attributes of the currently attended item are retrieved and integrated (Krajbich et al., 2010; but see Summerfield and Tsetsos, 2012; Calluso et al., 2015; for alternative drift diffusion models of value-based decision). At any point of time, the integrated value is then compared to the value of the unattended item. The agent freely explores the available options, switching their attention among the items. If the two items are appetitive (i.e., have been associated with positive experience in the past), the retrieval of their value will yield to a positive signal. While a specific item is being fixated, its value is computed and its relative value, compared to the other item, increases. When the difference between the values of the two items reaches a given threshold, the decision process terminates (Krajbich et al., 2010).

Evidence supporting this model is provided by experiments in perceptual decision-making (e.g., is the left segment shorter than the right one?) showing that in every choice, the firing rate of neurons increases proportionally to the easiness of the decision (integration process) and reaches the same point (threshold) right before an answer is given (Roitman and Shadlen, 2002; Gold and Shadlen, 2007). Moreover, during binary choices between snacks, the striatum and the ventromedial prefrontal cortex (i.e., two brain areas involved in valuation and choices) encode the value of the attended item, relatively to the value of the unattended item (Hare et al., 2011; Lim et al., 2011). Thus, attention modulates brain activity related to the retrieval and comparison of values.

The theory has several implications which have been verified experimentally. First, because the value of a desirable item increases when it is attended, the chosen item is the last one to be fixated before the threshold is reached and the decision is made. Second, the first fixated item gets an advantage in the value computation process and thus is more likely to be chosen. Third, the longer an item is being looked at the more likely it is that it will be chosen. Using repeated choices between snacks in combination with eye tracking, Krajbich et al. (2010) were able to confirm all those predictions. When choosing between two snacks equally liked by participants, they picked the last fixated item in about 75% of the trials. Moreover, the longest the first fixation, the higher the probability that the corresponding item would be chosen. Lastly, the longest an item was fixated and the higher was the probability it would be chosen, even after correcting for liking ratings. Importantly, similar choice biases induced by fixation trajectories were observed during purchasing decisions (Krajbich et al., 2012).

### Manipulating Attention to Bias Consumer Choices

As decision processes are strongly influenced by visual exploration, this evidence may imply that externally orienting attention would result in systematic decision biases. Indeed, controlling the duration of visual presentation of the options can change judgments about the attractiveness of human faces (Shimojo et al., 2003) and about moral situations (Pärnamets et al., 2015). Decisions to acquire food or art items (Armel et al., 2008; Lim et al., 2011)^1^ can be biased as well. The likelihood that an item is chosen increases between 6 and 11% when it was seen for 900 ms rather than 300 ms. Therefore, people have a bias to choose the things they have been viewing the longest rather than those they genuinely prefer. Gaze patterns reflect the preferences of individuals; they influence those preferences as well.

In addition, visually salient items would grab more attention (Itti and Koch, 2001), hence be fixated first and longer, and ultimately be chosen more often. Studies have shown that manipulating the visual saliency of stimuli by varying features such as intensity, color, and orientation results in participants making a choice that contradicts their initial preferences (Navalpakkam et al., 2010; Towal et al., 2013). These effects extend to purchasing environments, where they can become even stronger when the cognitive load is high. The color, and brightness of the packaging can lead individuals to choose their least preferred product under time pressure (Milosavljevic et al., 2012). Similarly, the probability that individuals will pick the brand they value the most in a supermarket shelf decreases as the number of available products increases. They tend to grab the product right in front of them. Because of reading habits, in occidental countries, options placed in the top left corner are chosen more often than those in lower right corner (Reutskaja et al., 2011).

### Applications in Marketing

Clearly, advertisers did not wait for psychologists and neuroscientists to describe the cognitive mechanisms of the attention grabbing effects on decisions to exploit them (Pieters and Wedel, 2004). Nonetheless as academic research makes progress in identifying decision biases, precisely describing the variables that can cause these biases in more and more refined theoretical models, advertising and other marketing techniques will become more effective. In fact, many efforts are directed into bridging neuroscience research with marketing both at the academic and at the industry levels (Plassmann et al., 2007; Karmarkar and Plassmann, 2019). Marketing companies are now equipped with a more mechanistic understanding of decisions processes and various neuroscientific tools to measure affective responses (skin conductance responses, pupil dilatation), attentional effects (eye movements, mouse movements), and brain responses elicited by products.

One particularly problematic ethical concern that derives from those new approaches is the ability to target specific individuals or groups of individuals (Stanton et al., 2017) via the systematic monitoring of consumers’ behavior, both online and in shops and the use of big data techniques to profile them (Aguirre et al., 2015; Boerman et al., 2017). The goal is to identify the putative needs of categories of consumers in order to focus the marketing strategy on selected goods susceptible to fill those needs. There are several risks associated with this practice, one being an increased consumerism and increased prices paid by consumers (Stanton et al., 2017). Another risk is to exploit the vulnerabilities of individuals. For instance, individuals, with compulsive buying disorders (Black, 2007) are particularly sensitive to encouragements to buy on the web (Rose and Dhandayudham, 2014). Marketing techniques can potentially have detrimental consequences on several groups of the population.

## Inter-Individual Differences in Decision-Making and Vulnerability to Marketing

Large inter-individual differences exist, both in decision mechanisms and their susceptibility to external influence. During development and aging, individuals tend to make less advantageous choices and are more susceptible to the influence of marketing techniques. Addiction and eating disorders can deeply tamper with the ability of making healthy choices. Recent advances in cognitive psychology and neuroscience can help understand why many individuals struggle in making sound choices.

### Children and Adolescents

Compared to adults, adolescents engage more in risky behavior (Steinberg, 2008) and display heightened peer-influence in their daily choices (van Hoorn et al., 2016). The uneven neurodevelopmental trajectories of the brain systems implicated in processing rewards on one side, and those involved in cognitive control on the other can explain these behavioral characteristics (Casey et al., 2008). The hyper-reactivity of the reward system, especially in the striatum is associated with emotional hypersensitivity to rewarding stimuli, faces and socio-emotional stimuli (Galvan et al., 2006; Casey et al., 2008; Hare et al., 2008). By contrast, the maturation of the prefrontal cortex, involved in cognitive control, still continues until about the age of 20 (Gogtay et al., 2004; Shaw et al., 2008).

Younger consumers constitute a substantial part of the market and marketers and advertisers have developed a large spectrum of strategies to reach them (Valkenburg and Cantor, 2001). The interest for marketing in children and adolescents lays in the realization that, in the last decades, they have acquired higher financial independence and more influence in household purchasing decisions. Children develop brand loyalty at an early age (Haryanto et al., 2016), which persists until adulthood. Detrimental effects of advertising on the development of children’s consumption habits is well documented (Wilcox et al., 2004). Television commercials targeted at children, in particular, are highly effective (Atkin, 1978; Gorn and Goldberg, 1982). They have been reported to induce unhealthy eating habits, to cultivate a materialistic value system and to be a source of conflicts between children and their parents (Goldberg and Gorn, 1978; Gorn and Goldberg, 1982; Story and French, 2004).

### Older Adults

Aging individuals constitute a particularly vulnerable population as well. Older individuals make more disadvantageous decisions, especially in uncertain or changing environments. One exception is the ability to make more farsighted decisions with age (Samanez-Larkin and Knutson, 2015) which can potentially lead to better consumer choices (Zauberman and Urminsky, 2016). However, older adults borrow at higher interest rates and pay more fees to financial institutions than their younger counterparts (Agarwal et al., 2007); they are less consistent in health-related decisions (Löckenhoff and Carstensen, 2007). Most importantly they are more sensitive to deceptive advertising than their younger counterparts (Denburg et al., 2007). Older adults’ heightened susceptibility to misleading advertising techniques can be explained by a reduced ability to discriminate between potentially misleading and more truthful advertising claims (Gaeth and Heath, 1987). They tend as well to give higher credit to claims that are repeated. Strikingly, even if they are informed that a claim is false, they will remember it as true a few days later (Skurnik et al., 2005). Decision deficits that arise with age in variable or uncertain environments might be due to cognitive limitations (Henninger et al., 2010; van de Vijver et al., 2015). Deficits in valuation processes have been also reported at the neural level, as structural changes in frontostriatal pathways are linked to disadvantageous decisions (Samanez-Larkin and Knutson, 2015; van de Vijver et al., 2016).

### Inter-Individual Differences in Self-Control

Individuals differ widely in their ability to implement self-control in their daily choices and maintain goal-directed behavior. Economists explain these disparities by considering inter-individual differences in discounting the long term consequences of choice options in the computation of their value (Laibson, 1997; O’Donoghue and Rabin, 1999). Psychologists approach this question by considering the relative difficulty and reliability of representing immediate pleasurable attributes and more abstract and temporally distant attributes of options (Liberman and Trope, 2008). When applied to self-control in dietary choices, eating a chocolate cake rather than an apple can be explained by the overweighing of taste compared to health information. A computational approach showed that up to 39% of the variability in dietary self-control failures can be explained by the speed with which the decision-making circuitry processes basic attributes like taste, versus more abstract attributes such as health (Sullivan et al., 2015). The biological plausibility of this model was supported by the finding that variability in diet success is linked to the relative representation of taste and health attributes in the ventromedial prefrontal cortex (Hare et al., 2009). According to the authors, “these findings provide a rationale for regulating marketing practices that increase the relative ease with which abstract attributes such as health are processed. For example, prominently displaying health information such as calorie counts may allow more rapid integration of health attributes” (Sullivan et al., 2015, p. 133).

In sum, the brain structures involved in motivation and decision-making are the latest to be fully functional during development and decline relatively early with age (Somerville and Casey, 2010; Samanez-Larkin and Knutson, 2015). As a result, maintaining goal-directed behavior in the long term and resisting temptations can be difficult at young age. Later in life, flexibly adapting to changing decision environments can become challenging (Eppinger et al., 2011). During adult life, unhealthy habits can readily form and several biological or societal factors can dysregulate the balance of the decision-making and motivation brain circuitry. Thus, large portion of the population is susceptible to be negatively impacted by marketing techniques and make disadvantageous decision or forming unhealthy habits, at least during certain period of their lives.

## Advertising Regulation

The realization of the increasing potential of neuroscientific knowledge applied to marketing raises a few questions. Does this always represent an advantage to us as a society and as individuals? If not, should (more) regulations be put in place to avert potential damage?

### Why Regulate Advertising?

In a world full of temptations carried by pervasive marketing messages, making decisions consistent with one’s own goals and preferences requires constant self-control. Extensive research has revealed that self-control often fails when individuals experience emotional distress (Baumeister et al., 1994). Excessive exposure to social norms brought by advertisement can induce emotional distress in vulnerable populations such as addicts or individuals with eating disorders. For instance, exposure to thin models in advertisement induces body-focused anxiety among women (Halliwell and Dittmar, 2004).

Research on the psychological consequences of poverty indicates a link between low income, stress and short-sighted, disadvantageous economical decisions (Haushofer and Fehr, 2014). In addition, financial scarcity causes a reduction in cognitive control (Mani et al., 2013), as well as changes in attention allocation; salient information relative to short-term decisions receive more attention than information concerning the future, which can cause bad economic decisions such as over-borrowing (Shah et al., 2012). Consequently, we might reasonably expect that poorer individuals can be negatively affected by advertising. While positive nudging can elicit people to save more (Karlan et al., 2016), tempting advertising or branding effects can easily lead to over-spending. Whether overexposure to marketing messages is linked to decreased well-being and increased level of stress or emotional distress in the general population is unknown, although some authors suggest it is likely to be the case (Baumeister, 2002; Sullivan et al., 2015). Research investigating this question is crucially needed in order to have a sound scientific dialogue about the “dark side of consumer neuroscience” (Kenning and Plassmann, 2008).

Internet advertising, in particular, potentially constitutes a serious concern. Internet ads are present in the visual field of consumers even when not directly attended. Several studies have shown that the value associated with specific stimuli are retrieved and updated by our reward system even when passively viewed (Lebreton et al., 2009; Tusche et al., 2010; Smith et al., 2014). Passive viewing of products of a specific brand have direct effect on purchase decisions (Ferraro et al., 2009). Additionally, with the generalization of online shopping, ads are present in the visual field of the buyer right at the moment of purchasing decisions. The use of internet data enables the tailoring of adverts by proposing to specific consumers those products they would be more likely to purchase. Online targeted advertising, through the monitoring of people’s online behavior triggers an increase in the rate of clicking on the ads as well as higher likelihood of purchase (Boerman et al., 2017), although the size of reported effects varies deeply between academic studies and claims made by advertising agencies.

### How to Regulate Advertising?

An efficient and self-regulated market rests on the ability for firms to inform consumers about their novel products and stimulate them to buy those products. Yet, this should not be done at the expense of individuals’ mental, physical or financial health. Neither should marketing strategies drive consumers away from their explicit goals and intentions, such as staying on a diet or reducing their use of products with high environmental impact. While people with strong initial preferences are less likely to see their choice behavior dramatically influenced by marketing techniques, the latter are more efficient on individuals whose preferences have not yet formed such as children, vulnerable groups or individuals with conflicting motivations.

We believe that expanding our knowledge about decision mechanisms and how to modulate them is not inherently problematic as many beneficial applications, for individuals and for the society, can arise. The rehabilitation of addictive disorders is one important application. Nudging, which can be considered as the ‘good’ counterpart to marketing, relies on very similar theories and techniques to influence individuals’ behavior to make it more in line with their intentions. One previously mentioned example is the use of reminders to save money. Another example is the so called ‘green-nudging’ (Schubert, 2017; Bonini et al., 2018) which prompts people to make ecologically responsible decisions. The key difference between marketing and nudging lies in the very idea of adequacy between the declared intentions of the customer (e.g., follow a specific diet, make ecologically responsible purchases) and the type of manipulation being exerted on their behavior. In addition, nudging is usually initiated by public institutions with the end goal of benefiting the society. For instance, nudging might encourage more ecologically responsible consumption by displaying the environment impact of products, but it will never orient consumers toward a specific brand. Public acceptability of nudging is generally positive (Reynolds et al., 2019) while advertising made by companies motivated by profit is controversial. Therefore, the very idea of transparency from the part of the advertising company and consent from the customer seems crucial. Policy makers could consider empowering citizens by letting them decide whether they accept to be exposed to different types of advertising.

Strikingly, the legal system of several countries has adjudicated that promoting products which threaten public health should be prohibited. Advertisement of products containing tobacco or alcohol is strictly forbidden in many countries. In addition, the branding effect of cigarettes is reduced by including pictures of dramatic health consequences of smoking on packaging. Similarly, attempts to reduce the prevalence of obesity, diabetes and hypertension have been made by trying to limit the effectiveness of advertisements on high caloric food and beverages with associated warning messages. For instance, in 2007 in France, a law was adopted listing categories of nutritive products (e.g., sweets and sodas) whose advertisement had to contain a message suggesting to eat more fruits and vegetable, increase physical activity and reduce salt and sugar intake. Thus, the approach adopted so far to protect the population from potential detrimental effects of advertising focuses on specific products and age groups (mainly children). Nonetheless, as discussed earlier the potential damage of advertising extent to many groups of individuals.

A possibly efficient approach could be to limit the intrusive aspects of the advertising means, in order to allow vulnerable individuals, especially those with compulsive or addictive tendencies, to maintain self-protective strategies. Measures should be taken to prevent advertisement to be forced into the peripheral visual field of individuals attending a nearby focal point of interest. In order to avoid passive viewing, it could entail the prohibition of advertising messages in confined public spaces (e.g., bus stops) and in locations surrounding informative or salient focal point (e.g., information panels). One particularly striking example is the advertisement low-cost airplane companies place on the seat in front of their clients to incite them to buy snacks. Such practice is extremely intrusive as people cannot easily look away. Similarly, if advertisement in magazines would be on their own separate page, rather than next to an informative article, consumers would still have the opportunity of being informed of new products while controlling the degree of exposure to advertisement they are willing to accept. Internet ads could be forced in their own browser tab instead of being placed next to the focus of attention of users. A mandatory op-out option for specific categories of products would also be desirable to help individuals struggling with addictive behavior or eating disorders. The important aspect in this proposition is to allow consumers to regain control in their exposure to advertisement by having them consent to viewing ads through a motor action (such as clicking on the ads tab), rather than forcing passive viewing.

## Conclusion

Due to our increasing knowledge of decision mechanisms and the increasing efficiency and outreach of communication means, marketing techniques are becoming both intrusive and powerful. The brain circuitry for decision and motivation changes during the lifespan or due to a diversity of contingent and individual factors. Because of our growing understanding of vulnerabilities to external influences, it is perhaps time to address the issue of intrusiveness of advertisement at a societal level and consider regulatory intervention.

## Author Contributions

NB and ER prepared and validated the manuscript.

## Conflict of Interest

The authors declare that the research was conducted in the absence of any commercial or financial relationships that could be construed as a potential conflict of interest.

## Footnotes

1 A demonstration of the effect is available in a TEDx talk delivered by Antonio Rangel (http://www.tedxcaltech.com/content/antonio-rangel).
